# Supplementary material for: Verifiable measurement-based quantum random sampling with trapped ions
Source: Nat Commun. 2025 Jan 2;16:106. doi: 10.1038/s41467-024-55342-3 (PMC11695993; doi:10.1038/s41467-024-55342-3)
Supplement: Supplementary file 1 — Supplementary Information [file 41467_2024_55342_MOESM1_ESM.pdf]

Supplementary Information for "Verifiable measurement-based quantum random sampling with trapped ions"

1. SUPPLEMENTARY NOTE 1 – FIDELITY WITNESS

In addition to the fidelity estimate, we have also measured a witness for the fidelity on the generalized cluster states [1, 2]. A fidelity witness of a quantum state $|\psi\rangle$ is a Hermitian operator W with the properties

$$\mathrm{Tr}[\rho W] = 1 \Leftrightarrow \rho = |\psi\rangle\langle\psi| \quad \text{and} \quad \mathrm{Tr}[\rho W] \leq F(\rho, |\psi\rangle). \tag{1}$$

In other words, the expectation value of a fidelity witness provides a meaningful lower bound on the fidelity of a state preparation ρ with a target state $|\psi\rangle$.

To construct a fidelity witness for the cluster states, we observe that the pre-measurement cluster state in the protocol is the ground state of a commuting, local Hamiltonian H with gap $\Delta = 2$, consisting of local terms given by the locally rotated stabilizers S_i of the cluster state. The energy of the local terms in an imperfect state preparation ρ yields a certificate for the fidelity $F = \langle\psi|\rho|\psi\rangle$ with the target state vector $|\psi\rangle$ in terms of the witness W_F as [1, 2]

$$W_F = 1 - \frac{1}{2}\sum_{i=1}^{N} \langle S_i\rangle_\rho \leq F. \tag{2}$$

In particular, this implies that the root infidelity bound (1) can be supplemented as

$$d_{\mathrm{TV}} \leq \sqrt{1-F} \leq \sqrt{1-W_F}. \tag{3}$$

We have measured the fidelity witness for the same quantum states prepared for Fig. 3 in the main text by measuring their stabilizers, see Fig. 1. We find that the upper bound (3) remains meaningful (i.e., smaller than one) only for very small sizes of the cluster, and conclude that the fidelity witness has very limited use in the presence of a significant amount of noise in the system.

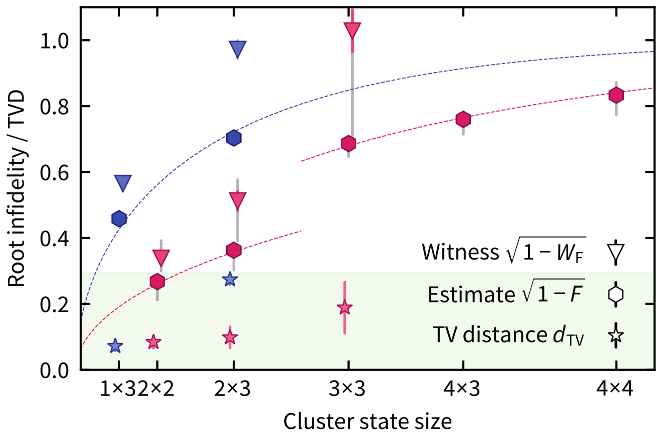

Supplementary Figure 1. **Experimental results for cluster states with and without recycling.** (Fig. 3 from the main text including the fidelity witness) Root infidelity witness $\sqrt{1-W_F}$ (triangles), root infidelity estimate $\sqrt{1-F}$ (hexagons), and empirical total-variation distance (stars) for single instances of random MBQC cluster states with recycling (blue) and without (pink). Colored error bars represent the 3σ interval of the statistical error. For uncorrelated measurement noise, the systematic measurement error only reduce the measured state fidelity. The worst-case behaviour of the measurement error is represented by the gray error bars. Modelling the circuit noise as local depolarizing noise after each entangling gate (dotted lines), we extract effective local depolarizing error probabilities of 5.3%, 2.6 % and 1% for the cluster with recycling, the cluster in the large register, and the cluster in the small register, respectively; see Eq. (44) below for details.

2. SUPPLEMENTARY NOTE 2 – EXPERIMENTAL DETAILS

In Table 1, we detail the number of experimental shots for sampling and verification used in Fig. 3 and Fig. 1.

	Size	Sampling shots	Verification shots		
			$M(W_F)$	$K(F)$	$M(F)$
Recycling	1 x 3	15 000	15 000	118 980	1
	2 x 3	62 500	3 600	223 980	1
Non-recycling	2 x 2	15 000	10 000	157 611	1
	2 x 3	15 000	2 500	154 177	1
	3 x 3	15 000	500	225 865	1
	4 x 3	—	50	110 408	1
	4 x 4	—	50	640	50

Supplementary Table 1. **Number of samples used in Fig. 3.** $M(W_F)$: Number of shots per stabilizer for the witness. $K(F)$: Number of randomly drawn stabilizers for the fidelity estimate. $M(F)$: Number of shots per stabilizer for the fidelity estimate.

3. SUPPLEMENTARY NOTE 3 – COMPILED CIRCUITS

The quantum circuit giving rise to the cluster state can be succinctly written as

$$|\mathrm{CS}(\beta)\rangle = \left(\prod_{k=1}^{N} \mathrm{e}^{-\mathrm{i}\beta_k Z_k}\right)\left(\prod_{\langle k,l\rangle} CZ_{k,l}\right)|+^N\rangle. \tag{4}$$

Here $\langle k, l\rangle$ denotes all pairs of neighboring ions in the respective cluster state. We express this circuit in terms of the native gates in the ion-trap architecture, pairwise addressed Mølmer Sørensen gates $\mathrm{MS}_{k,l}$ and arbitrary rotations around an axis in the X-Y plane $R(\theta,\phi)$, given by

$$\mathrm{MS}_{k,l} = \exp\left(-\mathrm{i}\frac{\pi}{4}X_k X_l\right), \tag{5}$$

$$R(\theta,\phi) = \exp\left(-\mathrm{i}\frac{\theta}{2}\left(\cos(\phi)X + \sin(\phi)Y\right)\right) = \begin{pmatrix} \cos\frac{\theta}{2} & -\mathrm{i}\mathrm{e}^{-\mathrm{i}\phi}\sin\frac{\theta}{2} \\ \mathrm{i}\mathrm{e}^{\mathrm{i}\phi}\sin\frac{\theta}{2} & \cos\frac{\theta}{2} \end{pmatrix}. \tag{6}$$

Here, the polar angle θ corresponds to the laser pulse area, while the azimuthal angle ϕ is determined by the phase of a laser pulse. With these definitions, we observe some properties of the rotation gate

- $X(\theta) = \mathrm{e}^{-\mathrm{i}\frac{\theta}{2}X} = R(\theta, 0)$,
- $Y(\theta) = R(\theta, \pi/2)$,
- $R(\theta, \phi+\pi)|0\rangle = \mathrm{e}^{\mathrm{i}\pi/2} Z R(\theta,\phi)|0\rangle$.

Since the phase can be controlled much more precisely than the pulse area, it is advantageous to only perform $\theta = \pi/2$-pulses with variable azimuthal angle ϕ. Now, let us decompose CZ in terms of the above gates. We start by writing

$$CZ_{k,l} = \mathrm{e}^{-\mathrm{i}\frac{\pi}{4}} \cdot \mathrm{e}^{\mathrm{i}\frac{\pi}{4}Z_i}\mathrm{e}^{\mathrm{i}\frac{\pi}{4}Z_j}\mathrm{e}^{-\mathrm{i}\frac{\pi}{4}Z_i Z_j}, \tag{7}$$

and then observe that $Y(\pi/2)Z(\pi) = H$, and since the rotation angle of Z does not matter for computational-basis measurements, we can replace H by $Y(\pi/2)$ and $H^\dagger = Z(-\pi)Y(-\pi/2)$. Hence $Y(-\pi/2)X(\theta)Y(\pi/2) = Z(\theta)$ such that

$$CZ_{k,l} = \mathrm{e}^{-\mathrm{i}\pi/4}\left(\prod_{i=k,l} Y_i(-\frac{\pi}{2})X_i(-\frac{\pi}{2})\right)\mathrm{MS}_{k,l}\left(\prod_{i=k,l} Y_i(\frac{\pi}{2})\right). \tag{8}$$

Putting this back into the first part of Eq. (4) we observe that intermediate $Y(\pm\pi/2)$ gates cancel and X-gates commute to the left leaving

$$\prod_{\langle k,l\rangle} CZ_{k,l} = \mathrm{e}^{-\mathrm{i}M\pi/4}\left(\prod_{i=1}^{N} Y_i\left(-\frac{\pi}{2}\right)X_i\left(-\frac{\deg(i)\pi}{2}\right)\right)\left(\prod_{\langle k,l\rangle}\mathrm{MS}_{k,l}\right)\left(\prod_{i=1}^{N} Y_i\left(\frac{\pi}{2}\right)\right), \tag{9}$$

where M is the total number of MS-gates and $\deg(i)$ is the degree of site i (i.e., the number of links in the cluster). We can further simplify

$$Z(\beta)H|0\rangle = Z(\beta+\pi)Y(\pi/2)|0\rangle = \mathrm{e}^{-\mathrm{i}\frac{\beta}{2}}R(\pi/2,\beta+\pi/2)|0\rangle = \mathrm{e}^{-\mathrm{i}(\frac{\beta}{2}-\frac{\pi}{2})}Z(\pi)R(\pi/2,\beta-\pi/2)|0\rangle, \tag{10}$$

and since $Y(\pi/2)Z(\pi) = X(\pi)Y(\pi/2)$ we obtain the total circuit

$$\begin{aligned}|\mathrm{CS}(\beta)\rangle &= \mathrm{e}^{-\mathrm{i}(M\pi/4+\frac{1}{2}\sum_i\beta_i - N\pi/2)}\left(\prod_{i=1}^{N} Y_i\left(-\frac{\pi}{2}\right)X_i\left(-\frac{(\deg(i)-2)\pi}{2}\right)\right)\left(\prod_{\langle k,l\rangle}\mathrm{MS}_{k,l}\right)\left(\prod_{i=1}^{N} Y_i\left(\frac{\pi}{2}\right)R_i\left(\frac{\pi}{2},\beta-\frac{\pi}{2}\right)\right) \\ &= \mathrm{e}^{-\mathrm{i}(M\pi/4+\frac{1}{2}\sum_i\beta_i - N\pi/2)}\left(\prod_{i=1}^{N} Y_i\left(-\frac{\pi}{2}\right)\left(X_i\left(-\frac{\pi}{2}\right)\right)^{\deg(i)-2}\right)\left(\prod_{\langle k,l\rangle}\mathrm{MS}_{k,l}\right)\left(\prod_{i=1}^{N} Y_i\left(\frac{\pi}{2}\right)R_i\left(\frac{\pi}{2},\beta-\frac{\pi}{2}\right)\right).\end{aligned} \tag{11}$$

To perform a measurement in the Hadamard basis, we can now rotate back using $H = ZY(-\pi/2)$, which can be absorbed in the leftmost layer of $Y(-\pi/2)$-gates, to give a layer of $Y(-\pi) = Y$ gates. Since these are just a phase flip and a bit flip, we can leave them out in the experiment and perform them in the classical postprocessing.

4. SUPPLEMENTARY NOTE 4 – COMPUTING THE THRESHOLD FIDELITY FROM STOCKMEYER'S ARGUMENT

In the rigorous argument for the hardness of quantum random sampling, one makes use of Stockmeyer's algorithm [3], an algorithm in the third level of the polynomial hierarchy, in order to estimate #P-hard probabilities; see Chapter 2 of Ref. [4] for an accessible explanation of the algorithm. Let us briefly summarize the argument here, and refer the reader to Ref. [5] for a more detailed exposition. We assume that there exists a classical algorithm $\mathcal{A}$ that samples from the output distribution p_U with probabilities $p_U(x) = |\langle x|U|0\rangle|^2$ up to an additive total-variation-distance error ε. We then feed $\mathcal{A}$ into Stockmeyer's algorithm and ask that it compute an approximation $q_U(x)$ of the probability $p_U(x)$ for some binary string x.

The crucial step in the hardness proof then consists in balancing the error stemming from Stockmeyer's algorithm itself and the error incurred from the assumption to obtain a multiplicative approximation up to a factor $1/4$ with constant probability over the choice of U and x. The relevant expression is given by applying Markov's inequality yielding that with probability $1-\delta$

$$|p_U(x) - q_U(x)| \leq \frac{p_U(x)}{\mathrm{poly}(n)} + \frac{\varepsilon}{2^n\delta}\left(1+\frac{1}{\mathrm{poly}(n)}\right). \tag{12}$$

We conjecture the distribution p_U to anticoncentrate in the sense that

$$\mathrm{Pr}_U\left[p_U(x) \geq \frac{1}{2^n}\right] \geq \gamma, \tag{13}$$

for some constant $\gamma > 0$. As a result we obtain that with probability $\gamma(1-\delta)$ Stockmeyer's algorithm yields a relative-error $\varepsilon/\delta + o(1)$ approximation of $p_U(x)$. Hence, assuming that any $\gamma(1-\delta)$-fraction of the instances is #P-hard to approximate up to relative error $\varepsilon/\delta + o(1)$, then this argument shows that one can approximate #P-hard quantities in the third level of the polynomial hierarchy – counter the established belief in theoretical computer science.

Consequently, we can trade the fraction of instances we conjecture to be hard with the tolerated error ε of the classical algorithm. Making a bolder average-case conjecture results in a larger error bound from the argument. As discussed in Ref. [6], we numerically find $\gamma = 1/\mathrm{e}$. Setting the average-case hard fraction $\nu = 10^{-3}$, and relative-error $\min(1-1/\sqrt{2}, \sqrt{2}-1) = 0.2928$ approximation [7], we obtain a threshold total variation-distance ν/γ and consequently threshold infidelity

$$1 - F_T = (0.2928\cdot(1-\nu/\gamma))^2 = 0.0857. \tag{14}$$

To summarize, we conjecture that any 10^{-3} fraction of the output probability instances is #P-hard to approximate. We then obtain that sampling is hard up to total-variation distance 0.29 with probability at least 1%. Correspondingly, the threshold infidelity of accepting a quantum state is roughly 8.6%; see Fig. 3.

We emphasize, however, that the threshold fidelity from the hardness argument above does not have a fundamental meaning in the sense that it does not imply easiness above the threshold. Rather, it gives a threshold such that—if the complexity-theoretic conjectures underlying the argument are true—hardness of sampling is guaranteed if the infidelity remains below the threshold. More generally, the takeaway should be that sampling from a family of distributions whose TVD to the target distributions does not increase if the cluster size is increased is likely a classically hard task; potentially even for a slow (polynomial) increase, see also Refs. [8, 9].

5. SUPPLEMENTARY NOTE 5 – PROPERTIES OF THE AVERAGE FIDELITY IN MEASUREMENT-BASED QUANTUM COMPUTING

In this section, we discuss the average fidelity

$$\overline{F} = \mathbb{E}_\beta[\langle \psi_\beta | \rho_\beta | \psi_\beta \rangle] \tag{15}$$

of state preparations ρ_β of the generalized cluster state $|\psi_\beta\rangle$ with respect to random local rotation angles $\beta \in [8]^{n\times m} \cdot \pi/4$.

First, in Section 5 A, we show that the *direct fidelity estimation* (DFE) protocol of Flammia and Liu [10] can be directly applied to estimating average state fidelities. Then, in Section 5 B we study properties of the cross-entropy measures in measurement-based computing, and in particular, discuss some specifics of measurement-based quantum computing, in particular, the role of logical and physical circuits for these cross-entropy measures. Given this, we show in Section 5 C how to use cross-entropy measures as an alternative way to estimate average fidelities, analogous to the theory of *cross-entropy benchmarking* (XEB) for random circuits by Arute *et al.* [11]. We show analytically that under certain assumptions on the noise in the device, such cross-entropy measures can indeed also be applied in the context of MBQC to estimate average fidelities. Lastly, in Section 5 D we support our analytical considerations with a numerical study comparing the resulting average fidelities from the DFE protocol with those obtained via XEB.

A. Direct average fidelity estimation

In this section, we show how DFE can be directly applied to estimating the average cluster state fidelity $\overline{F}$. To this end, we observe that, analogously to the single-instance case discussed in the Methods section of the main text,

$$\begin{aligned}\overline{F} &= \mathbb{E}_\beta\left[\frac{1}{2^N}\sum_{s_\beta\in\mathcal{S}_\beta}\langle s_\beta\rangle_{\rho_\beta}\right]\\ &= \frac{1}{2^N}\frac{1}{8^N}\sum_{\beta\in\frac{\pi}{4}\cdot[8]^N}\sum_{s_\beta\in\mathcal{S}_\beta}\sum_{\sigma=\pm1}\sigma\cdot\langle\pi^\sigma_{s_\beta}\rangle_{\rho_\beta}.\end{aligned} \tag{16}$$

Here, the stabilizer group $\mathcal{S}_\beta = \langle S_1^\beta, \ldots, S_N^\beta\rangle$ and $\pi^\sigma_{s_\beta}$ is the projector onto the σ-eigenspace of $s_\beta \in \mathcal{S}_\beta$. Hence, we obtain an unbiased estimator $\hat{\overline{F}}$ of the average fidelity by drawing a uniformly random pair (β, S_β), measuring S_β on ρ_β, and averaging over the measurement outcomes σ.

Since the measurement outcomes are bounded by 1 in absolute value, we can estimate the average up to error ϵ using a number M of uniformly random samples (β, S_β) that scales as $1/\epsilon^2$ and is in particular independent of the number of qubits. As before, if we measure M shots per random pair of state and stabilizer of which we draw K many, the variance of this will be given by the variance formula

$$\mathrm{Var}[\hat{\overline{F}}] = \frac{4}{KM}(\mathbb{E}_i[p_i](1-\mathbb{E}_i[p_i])) + \frac{4}{K}\left(1-\frac{1}{M}\right)\mathrm{Var}_i[p_i], \tag{17}$$

where the expectation runs over the choice of random state and stabilizer as labeled by i.

B. Cross-entropy benchmarking of measurement-based quantum computing

Cross-entropy benchmarking (XEB) has been proposed as a way to measure the average fidelity of quantum state preparations and has been applied to computations in the circuit model [11, 12], as well as to certain analog quantum simulations [13]. This benchmark has been developed by Arute *et al.* [11] and Boixo *et al.* [14], and the key property that makes it useful to experiments is that it can be sample-efficiently estimated for sufficiently random ensembles of circuits whose output probabilities are exponentially distributed. XEB has a complexity-theoretic interpretation in terms of the task dubbed *heavy outcome generation* [8, 15], an interpretation as a proxy for the TVD under assumptions on the noise in the classical output distribution [16], and provides a means to estimate the (average) quantum fidelity of the state from which the classical samples are produced [11], see Ref. [5] for an overview.

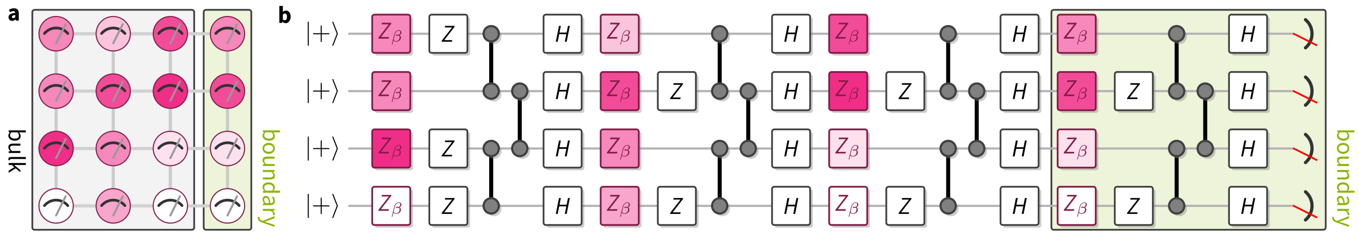

Supplementary Figure 2. **Distinction between physical and logical circuit.** **(a)** The *physical circuit* is the circuit which we apply in the experiment. It is a constant-depth circuit on $n \times m$ qubits that comprises a layer of single-qubit Hadamard gates and local rotations, and a layer of entangling CZ gates, followed by a measurement in the Hadamard basis. We further distinguish by convention the *bulk* of the lattice comprising the first $m-1$ columns and its *last column*. **(b)** The *logical circuit* is the effective depth-m circuit applied to the last column of qubits. It is generated by the measurements in the bulk of the cluster and, for each column of the physical state, comprises three layers: a layer of single-qubit rotations Z_β with rotation angles β corresponding to the respective position in the cluster and randomly applied Z gates depending on whether or not the outcome of the corresponding qubit was 1 or 0, a layer of entangling CZ gates, and a layer of Hadamard gates.

The most important XEB quantities are the so-called *linear* and *logarithmic XEB fidelities* (see, e.g., Ref. [5], Section V.B). For an ideal target probability distribution P and a noisy distribution Q on n-bit strings, we define those as

$$f_{\mathsf{lin}}(Q,P) := 2^n \sum_x Q(x)P(x) - 1, \tag{18}$$

$$f_{\mathsf{log}}(Q,P) := -\sum_x Q(x)\log P(x), \tag{19}$$

respectively. These quantities can be empirically estimated by drawing samples $x_1,\dots,x_k$ from the noisy distribution Q and averaging $P(x_1),\dots,P(x_k)$ or the logarithms of these, respectively.

For any single fixed circuit instance, the value of the XEB fidelities defined above and the actual quantum state fidelity of the state preparation before measurement could behave quite differently. A relation between XEB fidelity and quantum state fidelity can only be established on average over wide ensembles of circuits and only under additional assumptions described in more detail below. To this effect, we consider the average XEB fidelities $\overline{f}_{\mathsf{lin}}$ and $\overline{f}_{\mathsf{log}}$, where the average is taken over the ensembles of circuit instances. As described in Section 5 C, those average XEB fidelities will serve us as estimators of the average state fidelity $\overline{F}$.

We note that the above notions of XEB quantities apply to any model of quantum computation. In the following, we will discuss aspects of XEB that are particular to the measurement-based quantum computing setting considered in this work. We will denote the output probability distributions of an ideal cluster state preparation $|\psi_\beta\rangle$ and an imperfect cluster state preparation ρ_β upon measurement in the Hadamard basis, by P_β and Q_β, respectively. Now, in contrast to the circuit model, in the context of MBQC, we seem to face a choice regarding which distributions to base our XEB quantities on: Recall that in MBQC, the measurement outcomes in the 'bulk' of the system—that is, all qubits but those in the last (rightmost) column of the cluster—determine a *logical circuit* applied to the state $|+\rangle^{\otimes n}$ on the 'boundary' of the cluster—precisely that last column—see Fig. 2. According to this distinction between bulk and boundary, we split full outcome strings as $x = (x_b, x_f)$ into bulk outcomes $x_b \in \{0,1\}^{n(m-1)}$ and the outcomes on the final column $x_f \in \{0,1\}^n$.

Now, there are two output distributions that one could consider as inputs to XEB: First, there is the (joint) output distribution $P_\beta(x) = P_\beta(x_b, x_f)$ over the outcomes of the whole *physical circuit*, that is, the circuit which we actually apply in the lab, including all $N = n\cdot m$ measurement outcomes. Alternatively, there is the output distribution $\tilde{P}_{\beta,x_b}(x_f)$ of the logical circuit over the n outcomes x_f of only the last column of qubits. The logical circuit is determined by the choices of angles β as well as the $N-n$ measurement outcomes in the bulk x_b. Hence, the logical outcome distribution is simply the conditional distribution $\tilde{P}_{\beta,x_b} = P_\beta(\,\cdot\,|x_b)$ and the noisy samples are distributed according to $\tilde{Q}_{\beta,x_b} = Q_\beta(\,\cdot\,|x_b)$. This suggests, that we could also consider *logical* XEB quantities associated with the logical distribution $\tilde{P}_{\beta,x_b}(x_f)$ as follows

$$\tilde{f}_{\mathsf{lin}}(\tilde{Q}_{\beta,x_b},\tilde{P}_{\beta,x_b}) = 2^n \sum_{x_f} \tilde{Q}_{\beta,x_b}(x_f)\tilde{P}_{\beta,x_b}(x_f) - 1, \tag{20}$$

$$\tilde{f}_{\mathsf{log}}(\tilde{Q}_{\beta,x_b},\tilde{P}_{\beta,x_b}) = -\sum_{x_f} \tilde{Q}_{\beta,x_b}(x_f)\log \tilde{P}_{\beta,x_b}(x_f). \tag{21}$$

A motivation for considering logical output distributions of an $n \times m$ cluster state is that, in contrast to the physical output distributions, the logical ones behave analogously to the outcome distribution of random circuits on n qubits with depth

$O(m)$. In particular, their statistical properties—which are an important ingredient in XEB theory—match up to constant factors [17]. Conversely, if our goal is to estimate the average fidelity of the full cluster state, the XEB fidelity of the physical output distribution $P_\beta(x_b, x_f)$ seems to be the relevant quantity.

As it turns out, however, the XEB fidelities associated with physical and logical output distributions are in fact equivalent when used for average fidelity estimation via XEB. To see this, we have to consider the averaged XEB quantities introduced above. Concretely, we consider averages over the ensemble of circuits induced by a uniformly random choice of β from $\frac{\pi}{4} \cdot [8]^{n\times m}$. This random choice induces via the map $\beta \mapsto P_\beta$, a distribution over ideal output distributions which we denote by $\mathcal{P}$. Similarly, we denote by $\mathcal{Q}$ the distribution over noisy output distributions induced via $\beta \mapsto Q_\beta$. Hence, the average XEB fidelities are written as

$$\overline{f}_{\mathsf{lin}}(\mathcal{Q}, \mathcal{P}) = \mathbb{E}_{Q\sim\mathcal{Q}, P\sim\mathcal{P}}\left[f_{\mathsf{lin}}(Q, P)\right], \tag{22}$$

$$\overline{f}_{\mathsf{log}}(\mathcal{Q}, \mathcal{P}) = \mathbb{E}_{Q\sim\mathcal{Q}, P\sim\mathcal{P}}\left[f_{\mathsf{log}}(Q, P)\right]. \tag{23}$$

Analogously, the average logical XEB fidelities arise from drawing logical circuit instances (β, x_b) according to the uniform choice of β and x_b from the marginal distribution with probabilities $Q_\beta(x_b) = \sum_{x_f} Q_\beta(x_f, x_b)$.

Now, to relate the average XEB fidelites corresponding to physical and logical circuits, we will use the fact that, under the ideal distribution $P_\beta(x)$, the outcomes x_b are uniformly distributed [18] so that $P_\beta(x_f, x_b) = 2^{-n(m-1)} P_\beta(x_f|x_b)$. Then, we compute

$$\begin{aligned}
\mathbb{E}_\beta\left[f_{\mathsf{lin}}(Q_\beta, P_\beta)\right] &= 2^N \mathbb{E}_\beta \sum_x Q_\beta(x) P_\beta(x) - 1, \\
&= 2^N \mathbb{E}_\beta \sum_{x_b} \sum_{x_f} Q_\beta(x_f, x_b) P_\beta(x_f, x_b) - 1, \\
&= 2^n \mathbb{E}_\beta \sum_{x_b} Q_\beta(x_b) \sum_{x_f} Q_\beta(x_f|x_b) P_\beta(x_f|x_b) - 1, \\
&= 2^n \mathbb{E}_\beta \mathbb{E}_{x_b} \sum_{x_f} \tilde{Q}_{\beta,x_b}(x_f) \tilde{P}_{\beta,x_b}(x_f) - 1, \\
&= \mathbb{E}_\beta \mathbb{E}_{x_b}\left[\tilde{f}_{\mathsf{lin}}(\tilde{Q}_{\beta,x_b}, \tilde{P}_{\beta,x_b})\right],
\end{aligned} \tag{24}$$

which we can rewrite in short notation as

$$\overline{f}_{\mathsf{lin}} = \overline{\tilde{f}}_{\mathsf{lin}}. \tag{25}$$

An analogous computation yields

$$\overline{f}_{\mathsf{log}} = \overline{\tilde{f}}_{\mathsf{log}} + \log 2^{n(m-1)}. \tag{26}$$

To summarize, the physical and logical average XEB fidelities are equivalent in both the linear and the logarithmic versions up to a shift for the log XEB fidelity. However, their empirical variance—given in Eq. (59)—might still differ, because the samples are grouped differently in the mean of means estimator. In practice, we will therefore use XEB estimates from the physical circuits whenever possible given system size constraints (which influences the complexity of computing the ideal probabilities). This is because we are able to take more samples per circuit (reducing the first term of Eq. (59)) since the circuits of the logical XEB fidelity are partially determined by the—random—physical measurement outcomes. Moreover, in the experimental setting, taking more samples per circuit is cheaper than running more different circuits.

Ideal values of the XEB fidelity. For completeness, we conclude this subsection by demonstrating how to compute *ideal values* of the average XEB fidelities. These are the values of $\overline{f}_{\mathsf{lin}}$ and $\overline{f}_{\mathsf{log}}$ resulting from the case where $Q_\beta = P_\beta$, i.e.

$$\overline{f}_{\mathsf{lin}}(\mathcal{P}, \mathcal{P}) = \mathbb{E}_\beta\left[f_{\mathsf{lin}}(P_\beta, P_\beta)\right] \quad \text{and} \quad \overline{f}_{\mathsf{log}}(\mathcal{P}, \mathcal{P}) = \mathbb{E}_\beta\left[f_{\mathsf{log}}(P_\beta, P_\beta)\right]. \tag{27}$$

To compute these ideal values we make use of statistical properties of the logical output distributions. In particular, it was shown by Haferkamp *et al.* [17] that the logical circuits form an ϵ-approximate 2-design in depth $m \in O(n + \log(1/\epsilon))$. This implies that the second moments $\mathbb{E}_{\beta,x_b}[\tilde{P}^2_{\beta,x_b}]$ of the ideal logical output probability distributions approximate the Haar-random value with relative error ϵ. Neglecting this error, we find that the ideal average linear XEB fidelity of circuits with such scaling of m

with n asymptotically behaves as

$$\begin{aligned}\overline{f}_{\text{lin}}(\mathcal{P},\mathcal{P}) &= \overline{\overline{f}}_{\text{lin}}(\mathcal{P},\mathcal{P}),\\ &= 2^n \sum_{x_f} \mathbb{E}_{\beta,x_b}\left[\tilde{P}_{\beta,x_b}(x_f)^2\right] - 1,\\ &= \frac{2\cdot 2^{2n}}{2^n(2^n+1)} - 1,\\ &= \frac{2}{1+2^{-n}} - 1 \approx 1 - \frac{1}{2^{n-1}}.\end{aligned} \tag{28}$$

In particular, because of the equality of physical and logical linear XEB, the ideal value can only depend on the size of the logical circuit which is in turn given by the shortest side of the square lattice. Moreover, it is easy to see that the average linear XEB fidelity with the uniform distribution $\mathcal{U}$ (defined by $\beta \mapsto U([2^n]) := (2^{-n}, 2^{-n}, \ldots, 2^{-n}) \in [0,1]^{2^n}$) is given by $\overline{f}_{\text{lin}}(\mathcal{U},\mathcal{P}) = 0$.

We can repeat the same calculation for the logarithmic XEB fidelity, to find

$$\begin{aligned}\overline{f}_{\text{log}}(\mathcal{P},\mathcal{P}) &= \overline{\overline{f}}_{\text{log}}(\mathcal{P},\mathcal{P}) + \log 2^{n(m-1)},\\ &= -\sum_{x_f} \mathbb{E}_{\beta,x_b}\left[\tilde{P}_{\beta,x_b}(x_f)\log \tilde{P}_{\beta,x_b}(x_f)\right] + \log 2^{n(m-1)}, &(29)\\ &= \log 2^n - 1 + \gamma + \log 2^{n(m-1)}, &(30)\\ &= \log 2^{nm} - 1 + \gamma\end{aligned}$$

where the step from Eq. (29) to (30) follows from the properties of the exponential distribution (see Ref. [14, Sec. II of the SI] for details) and $\gamma \approx 0.5774$ is the Euler constant. In particular, this calculation implies that for distributions with uniformly distributed marginals and Porter-Thomas distributed conditional probabilities, we get the same average value as for global Porter-Thomas distributed distributions. Likewise, we find the average value of the log XEB fidelity when comparing it to the uniform distribution $\mathcal{U}$ to be

$$\overline{f}_{\text{log}}(\mathcal{U},\mathcal{P}) = -\mathbb{E}_\beta[\log P_\beta(x)] = \log 2^{nm} + \gamma, \tag{31}$$

again, assuming Porter-Thomas shape of the distribution.

The theoretical ideal values found above pertain to the asymptotic limit of cluster states of increasing size. However, in the experiments reported in the main text, we deal with small instance sizes. In Fig. 3, we confirm the convergence to the ideal values computed above for small instance sizes as relevant to our experiment. We find that the linear XEB fidelity significantly deviates from the expected asymptotic value for small sizes $n \times m$ of the cluster. It is also true that the XEB fidelity of $n \times m$ clusters equals that of $m \times n$ clusters and is given by the ideal value of the logical circuit corresponding to the shorter side. When we measure the deviation of the ideal XEB fidelity of the logical circuits from their expected value, this deviation decays exponentially with the depth m of the logical circuit (corresponding to the width of the cluster), independently of the number of qubits n (corresponding to its height)—until we hit the noise floor set by the precision of our computation around $m = 10$. Note that in this comparison, we sometimes compare the XEB fidelities of rectangular clusters to the ideal XEB fidelity corresponding to the *longer side*, namely whenever $n \geq m$.

In contrast, the ideal logarithmic logical XEB is almost immediately close to its ideal value (24), but the deviation (after an initial decay) stays roughly constant with the width of the cluster while it decays with the height. We interpret this fact in terms of the Porter-Thomas distribution: For a small number of qubits n on which the logical circuit acts, the exponential distribution is not a good approximation of the actual distribution of output probabilities of Haar-random quantum states. Hence, the calculation of the mean value, which uses the exponential distribution, incurs a systematic error. See the Supplementary Material of Ref. [19] for details of this calculation. Finally, the deviation of the ideal log XEB fidelity for uniform samples from the expected values decay with both the height and the width of the cluster. For the small-size experiments (up to $m = n = 4$) we therefore need to use the computed values of the XEB fidelities (instead of the asymptotic values) when using XEB to estimate the fidelity; see the subsequent section.

C. Estimating the average fidelity via XEB

In this section, we discuss how the average XEB fidelities $\overline{f}_{\text{lin}}$ and $\overline{f}_{\text{log}}$ and their logical counterparts $\overline{\overline{f}}_{\text{lin}}$ and $\overline{\overline{f}}_{\text{log}}$ can be used to estimate the average state fidelity $\overline{F}$ of the underlying quantum states under assumptions on the noise in the device. We follow the argumentation of Arute *et al.* [11, Sec. IV.A, SI], see also Ref. [5, Sec. V.B.3] for an overview.

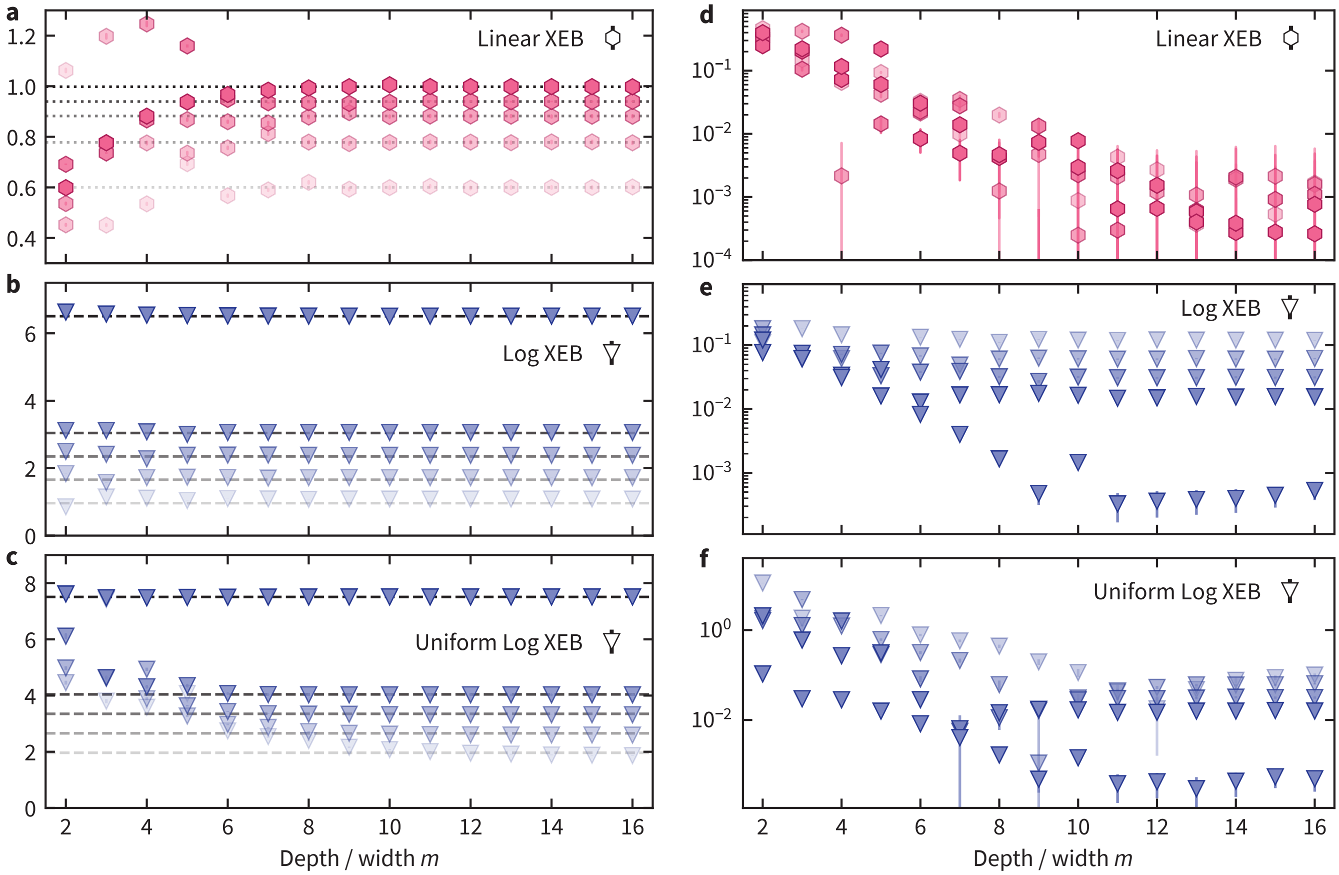

Supplementary Figure 3. **Ideal values of the linear and logarithmic XEB fidelities.** We compute the ideal values of the XEB fidelities for clusters of size $n \times m$ with fixed values of the height $n = 2, 3, 4, 5, 10$ (corresponding to the number of qubits of the logical circuit) in increasing opacity and scaling of the width m (corresponding to the depth of the logical circuit). To this end, we average the XEB fidelities of the ideal output distributions of $K = 10^5$ random (logical) circuits per data point. Error bars are 3σ intervals. (a) Ideal values of the linear XEB (pink hexagons). For the linear XEB fidelity, the ideal value of the physical circuit equals that of the logical circuit by the uniformity property of the marginals, cf. Eq. (24). Dotted lines indicate the ideal values (28). (b) Ideal value of the logical log XEB fidelity $\overline{\overline{f}}_{\mathsf{log}}(\mathcal{P}, \mathcal{P})$ (blue triangles). Dashed lines represent the asymptotic value $\log 2^n + \gamma - 1$ (Eq. (30) & (26)). (c) Logical log XEB fidelity for uniform samples $\overline{\overline{f}}_{\mathsf{log}}(\mathcal{U}, \mathcal{P})$. Dashed lines indicate the ideal value $\log 2^n + \gamma$ (Eq. (31)). (d–f) Deviations of the estimated finite-size values from the ideal values of (a–c), respectively.

1. *Linear XEB fidelity with depolarizing noise*

Let us begin with the linear XEB fidelity. We consider first the toy model of global depolarizing noise and then generalize it to uncorrelated and unbiased noise.

Depolarizing noise. Consider the noisy state

$$\rho_\beta(\epsilon) = \epsilon|\psi_\beta\rangle\langle\psi_\beta| + (1-\epsilon)\mathbb{1}/2^N. \tag{32}$$

where $|\psi_\beta\rangle$ is the generalized cluster state. Then, the fidelity is given by $F = \langle\psi_\beta|\rho_\beta(\epsilon)|\psi_\beta\rangle = \epsilon + (1-\epsilon)/2^N$, and the same holds true for the average fidelity $\overline{F}$. In this case, the XEB fidelity is given by

$$f_{\mathsf{lin}}(Q_\beta(\epsilon), P_\beta) = \epsilon f_{\mathsf{lin}}(P_\beta, P_\beta) + (1-\epsilon) f_{\mathsf{lin}}(U([2^N]), P_\beta). \tag{33}$$

Averaging over β, we find

$$\begin{aligned} \overline{f}_{\mathsf{lin}}(\mathcal{Q}, \mathcal{P}) &= \mathbb{E}_\beta[f_{\mathsf{lin}}(Q_\beta(\epsilon), P_\beta)] \\ &= \epsilon\, \mathbb{E}_\beta[f_{\mathsf{lin}}(P_\beta, P_\beta)] + (1-\epsilon)\underbrace{\overline{f}_{\mathsf{lin}}(\mathcal{U}, \mathcal{P})}_{=0} \\ &= \epsilon \overline{f}_{\mathsf{lin}}(\mathcal{P}, \mathcal{P}), \end{aligned} \tag{34}$$

and hence, we can estimate ϵ as

$$\hat{\epsilon} = \frac{\hat{\overline{f}}_{\mathsf{lin}}(\mathcal{Q},\mathcal{P})}{\overline{f}_{\mathsf{lin}}(\mathcal{P},\mathcal{P})}, \tag{35}$$

where $\hat{\overline{f}}_{\mathsf{lin}}(\mathcal{Q},\mathcal{P})$ is the empirical estimate of the experimental average linear XEB fidelity. We can then estimate the average fidelity as

$$\overline{F}(\hat{\epsilon}) = \hat{\epsilon} + (1-\hat{\epsilon})/2^{nm}. \tag{36}$$

Arute *et al.* [11] justify this estimator further using Bayes rule.

Uncorrelated and unbiased noise. One can make the same argument in case the quantum state is given by some noisy state

$$\rho_\beta(\epsilon) = \epsilon|\psi_\beta\rangle\langle\psi_\beta| + (1-\epsilon)\chi_\beta, \tag{37}$$

decomposed into the ideal state and a state χ_β capturing the noise. Now, the same conclusions regarding estimates of the *average fidelity* will hold in case the noise is

- *uncorrelated* in the sense that $\mathbb{E}_\beta[\langle x|\chi_\beta|x\rangle\langle x|\psi_\beta\rangle\langle\psi_\beta|x\rangle] = \mathbb{E}_\beta[\langle x|\chi_\beta|x\rangle]\,\mathbb{E}_\beta[\langle x|\psi_\beta\rangle\langle\psi_\beta|x\rangle]$, and
- *unbiased* in the sense that $\mathbb{E}_\beta[\langle x|\chi_\beta|x\rangle] = 1/2^N$.

The estimator $\hat{\overline{F}}$ using Eq. (35) will then give a good estimate of the average fidelity.

We note that one can similarly relate the average logical linear XEB $\overline{\tilde{f}}_{\mathsf{lin}}$ to the average fidelity of the "logical" output state, i.e., the state on the final column that arises from measuring the bulk qubits obtaining some outcome x_b which we denote by $|\psi_{\beta,x_b}\rangle$. To see this, consider this noisy logical state $\rho_{\beta,x_b}(\tilde{\epsilon})$ and write analogously to Equation (32)

$$\rho_{\beta,x_b}(\tilde{\epsilon}) = \tilde{\epsilon}|\psi_{\beta,x_b}\rangle\langle\psi_{\beta,x_b}| + (1-\tilde{\epsilon})\mathbb{1}/2^n. \tag{38}$$

Then, the average fidelity of the noisy logical output state is given by

$$\overline{\tilde{F}}(\tilde{\epsilon}) = \tilde{\epsilon} + (1-\tilde{\epsilon})/2^n. \tag{39}$$

In analogy to Equation (35), this average logical fidelity can be estimated via the average logical XEB as follows

$$\hat{\tilde{\epsilon}}_{\mathsf{lin}} = \frac{\hat{\overline{\tilde{f}}}_{\mathsf{lin}}(\mathcal{Q},\mathcal{P})}{\overline{\tilde{f}}_{\mathsf{lin}}(\mathcal{P},\mathcal{P})} = \hat{\epsilon}_{\mathsf{lin}} \tag{40}$$

where the last equality follows from the equivalence of physical and logical average linear XEB derived in Equation (25).

2. Log-XEB

We can repeat the same argument for the logarithmic XEB under the global depolarizing noise assumption, and find

$$\overline{f}_{\mathsf{log}}(\mathcal{Q},\mathcal{P}) = \mathbb{E}_\beta[f_{\mathsf{log}}(Q_\beta(\epsilon), P_\beta)] \tag{41}$$

$$= \epsilon\overline{f}_{\mathsf{log}}(\mathcal{P},\mathcal{P}) + (1-\epsilon)\overline{f}_{\mathsf{log}}(\mathcal{U},\mathcal{P}) \tag{42}$$

and hence an estimator of ϵ is given by

$$\hat{\epsilon}_{\mathsf{log}} = \frac{\hat{\overline{f}}_{\mathsf{log}}(\mathcal{Q},\mathcal{P}) - \overline{f}_{\mathsf{log}}(\mathcal{U},\mathcal{P})}{\overline{f}_{\mathsf{log}}(\mathcal{P},\mathcal{P}) - \overline{f}_{\mathsf{log}}(\mathcal{U},\mathcal{P})}. \tag{43}$$

Again, from $\hat{\epsilon}$, we can estimate the average (physical) fidelity according to $\overline{F}(\hat{\epsilon})$. Again, we find $\hat{\epsilon}_{\mathsf{log}} = \hat{\tilde{\epsilon}}_{\mathsf{log}}$, since all logical logarithmic XEB quantities are just shifted by $\log 2^{n(m-1)}$. However, for the same derivation to work with more general noise, we need to adapt the "uncorrelated" assumption to the logarithm, i.e., $\mathbb{E}_\beta[\langle x|\chi_\beta|x\rangle \log(P_\beta(x)] = \mathbb{E}_\beta[\langle x|\chi_\beta|x\rangle]\,\mathbb{E}_\beta[\log(P_\beta(x)]$, while the unbiasedness condition remains the same. Notice that all estimators above are unbiased since they are just linear in the empirical estimates of the XEB fidelities.

3. How to estimate fidelity

While the estimates $\hat{\hat{\epsilon}}$ and $\hat{\epsilon}$ always agree, the average fidelity estimators thus differ in the normalization of the correction term to the state fidelity in Eqs. (36) and (39). Which correction to the average depolarizing fidelity will yield a better estimate of the fidelity depends on how accurate the uncorrelated and unbiased noise assumption is for the logical versus the physical output state, or in other words, how well the model of Eqs. (32) and (37) applies to the corresponding states.

Dalzell *et al.* [20] show that for local random circuits of at least logarithmic depth, local depolarizing noise approximately transforms into global depolarizing (white) noise at the level of the output state and thus build confidence in the validity of these assumptions. Specifically, Dalzell *et al.* [20] prove that the white-noise assumption is approximately true in random circuits provided the physical noise is local and unbiased. In that case, the effective noise at the end of the circuit will be approximately depolarizing with an error scaling inversely with the number of gates. More precisely, they show that the normalized linear XEB $\overline{f} = \overline{f}_{\mathsf{lin}}(\mathcal{Q}, \mathcal{P})/\overline{f}_{\mathsf{lin}}(\mathcal{P}, \mathcal{P})$ between the noisy distribution $\mathcal{Q}$ and the ideal distribution $\mathcal{P}$ behaves as

$$\overline{f}(\eta) = \mathrm{e}^{-2S\eta}, \tag{44}$$

where S is the number of two-qubit gates and η is the probability of a local Pauli error on each qubit after a two-qubit gate. Moreover, for incoherent *unital* noise, the noisy distribution approaches the uniform distribution at the same rate with an error given by $O(\overline{f}_{\mathsf{lin}}\eta\sqrt{S})$ all in the regime of $S \in \Omega(n \log n)$.

Given that the statistical properties of random logical MBQC circuits behave completely analogously to those of random circuits in the circuit model, we would expect an analogous result to hold for the fidelity of the logical output state in MBQC. More precisely, random logical MBQC circuits behave like random universal circuits on the level of lower moments in the sense that random logical MBQC circuits also generate unitary 2-designs [17] (and presumably polynomial designs as well). A possible caveat, however, is that in MBQC physical noise translates non-trivially into logical noise as considered for single-qubit circuits by Usher and Browne [21]. Thus, while we are unclear on the exact conditions on a local noise model, we do expect that an analogous result to that of Dalzell *et al.* [20] holds for logical MBQC circuits. In this case, the logical XEB fidelity will be a good measure of the quantum fidelity of the output of the logical circuit. In this case, the fidelity will decay approximately according to Eq. (44).

Notice, though, that our direct estimate of the fidelity measures the fidelity of the *physical output state* and hence we cannot experimentally certify that XEB yields quality estimates for the logical fidelity $\overline{\overline{F}}$. But for the physical output state, we are much less confident in the validity of the uncorrelatedness and unbiasedness of the noise with the circuit. Indeed, a priori, there is no good reason to expect that the physical XEB fidelity estimator $\overline{F}(\hat{\epsilon})$ matches the physical fidelity $\overline{F}$ accurately unless physical and logical average fidelity behave in the same way.

We do find, however, that the estimator $\overline{F}(\hat{\epsilon})$ works reasonably well as an estimate for the fidelity for local depolarizing noise, and also the noise we face in the experiment; see the following section. This suggests that the uncorrelated and unbiasedness assumption does in fact hold true for the physical circuit as well. Furthermore, it suggests, that as n, m grow, the physical and logical physical fidelity converge. We leave a more detailed analysis of the effect of noise in MBQC on the estimates of fidelity to future work. In the following section, we will provide numerical evidence that, indeed, the XEB fidelities can be used to estimate the quantum fidelity in the presence of various types of local noise on the physical circuit.

D. A numerical study

In the previous sections, we have explained how to obtain estimates $\hat{\overline{F}}$ for the average fidelity of the state preparation of the cluster state in two different ways:

- From random stabilizer measurements on the cluster state via DFE as considered in Section 5 A.
- From quantum random sampling in the Hadamard basis via XEB fidelity estimation as explained in Section 5 C.

In this section, we numerically study the quality of fidelity estimates obtained via XEB fidelity estimation by using the estimates obtained via DFE as a benchmark. In particular, we generate data according to the above-mentioned two methods by numerically simulating noisy state preparations ρ_β of the cluster state for many randomly drawn β. We do so for different types of noise, system sizes, and noise strengths. From these data, we then obtain the corresponding average fidelity estimates as a function of the system size and noise strength. More concretely, we numerically simulated two different settings.

The first setting is inspired by the theoretical work of Dalzell *et al.* [20] on random circuit sampling under local, unbiased noise. This is the setting in which we most likely expect the average XEB fidelity estimate $\overline{F}(\hat{\epsilon})$ to be a good estimate of the average fidelity $\overline{F}$. Here, we consider noisy cluster state preparations ρ_β via circuits built from Hadamard and CZ gates and Z-rotation gates. We take all single-qubit gates to be perfect but all CZ gates are followed by local depolarizing or dephasing noise channels, respectively. In Fig. 4 and Fig. 5 we compare the fidelity estimates obtained via the DFE and XEB methods for

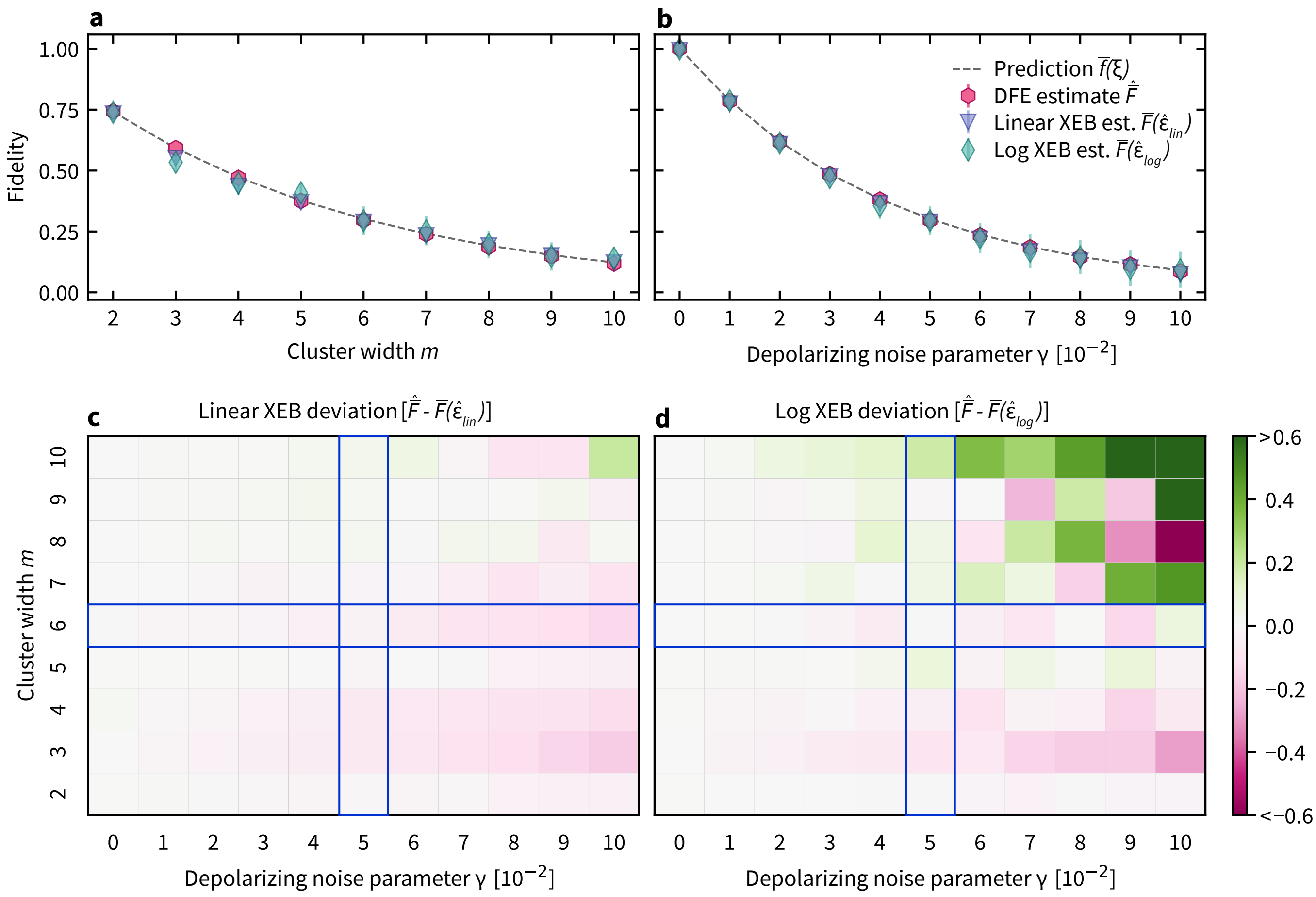

Supplementary Figure 4. **Average fidelity estimation for depolarizing noise.** We simulate sampling in the Hadamard basis and DFE for noisy random cluster states of size $2 \times m$ for $m \in \{2, 3, \ldots, 10\}$ with local depolarizing noise with parameter γ after every entangling CZ gate. We sample $K = 10^5$ random circuits and take $M = 50$ samples per circuit. From the classical samples, we compute estimates of the average fidelity $\overline{F}(\hat{\epsilon})$ according to (36) using the linear and logarithmic average XEB fidelity $\overline{f}_{\text{lin}}$ and $\overline{f}_{\text{log}}$ (represented by purple triangles and green diamonds, respectively) and compare them to the DFE estimate $\hat{\overline{F}}$ of the average fidelity (pink hexagons) as well as the prediction (44) by Dalzell *et al.* [20] (dotted line). Error bars are 3σ intervals. (a) Decay of the average fidelity with the width of the cluster state for fixed noise parameter $\gamma = 0.05$. (b) Decay of the average fidelity with the noise parameter for a fixed width of the cluster $m = 6$ (blue boxes). (c) Deviation $\overline{F}(\hat{\epsilon}_{\text{lin}}) - \hat{\overline{F}}$ between the fidelity estimate from the linear XEB fidelity and the average fidelity. (d) Deviation $\overline{F}(\hat{\epsilon}_{\text{log}}) - \hat{\overline{F}}$ between the fidelity estimate from the linear XEB fidelity and the average fidelity.

these two noise settings. We also compare these results to fidelity scaling predicted via the formula (44), where the number of two-qubit CZ gates is in our case just the number of edges of the $n \times m$ square lattice, given by $n(m-1) + m(n-1)$. Writing the single-qubit depolarizing and dephasing channels $\mathcal{D}_\gamma$ and D_ξ with parameters γ and ξ, respectively, as

$$\mathcal{D}_\gamma(\rho) = (1-\gamma)\rho + \gamma\frac{\mathbb{1}}{2} \tag{45}$$

$$D_\xi(\rho) = (1-\gamma)\rho + \xi\,\mathrm{diag}(\rho), \tag{46}$$

the error probability η takes values $\eta = 3\gamma/4$ for depolarizing noise and $\eta = \xi/2$ for dephasing noise.

We find excellent agreement of the prediction $\overline{F}(\eta)$ for the average physical fidelity—although it was derived for the XEB fidelity, while the XEB fidelity estimators are approximately correct for depolarizing noise in the regime of low noise parameters $\gamma \sim 1/n$. For dephasing noise, we find that the XEB fidelity estimators greatly underestimate the average fidelity.

In contrast, the second setting models the actual experimental setup reported in the main text. That is, we simulate the noisy experimental circuits described in the Methods section. Again, we find excellent agreement of the physical fidelity with the prediction $\overline{F}(\eta)$ by Dalzell *et al.* [20]. We find that setting the effective depolarizing noise parameter $\gamma = 1 - \exp(-0.310\sigma^2/2)$, where σ is the measure of the noise strength, gives the best fit with the observed fidelity.

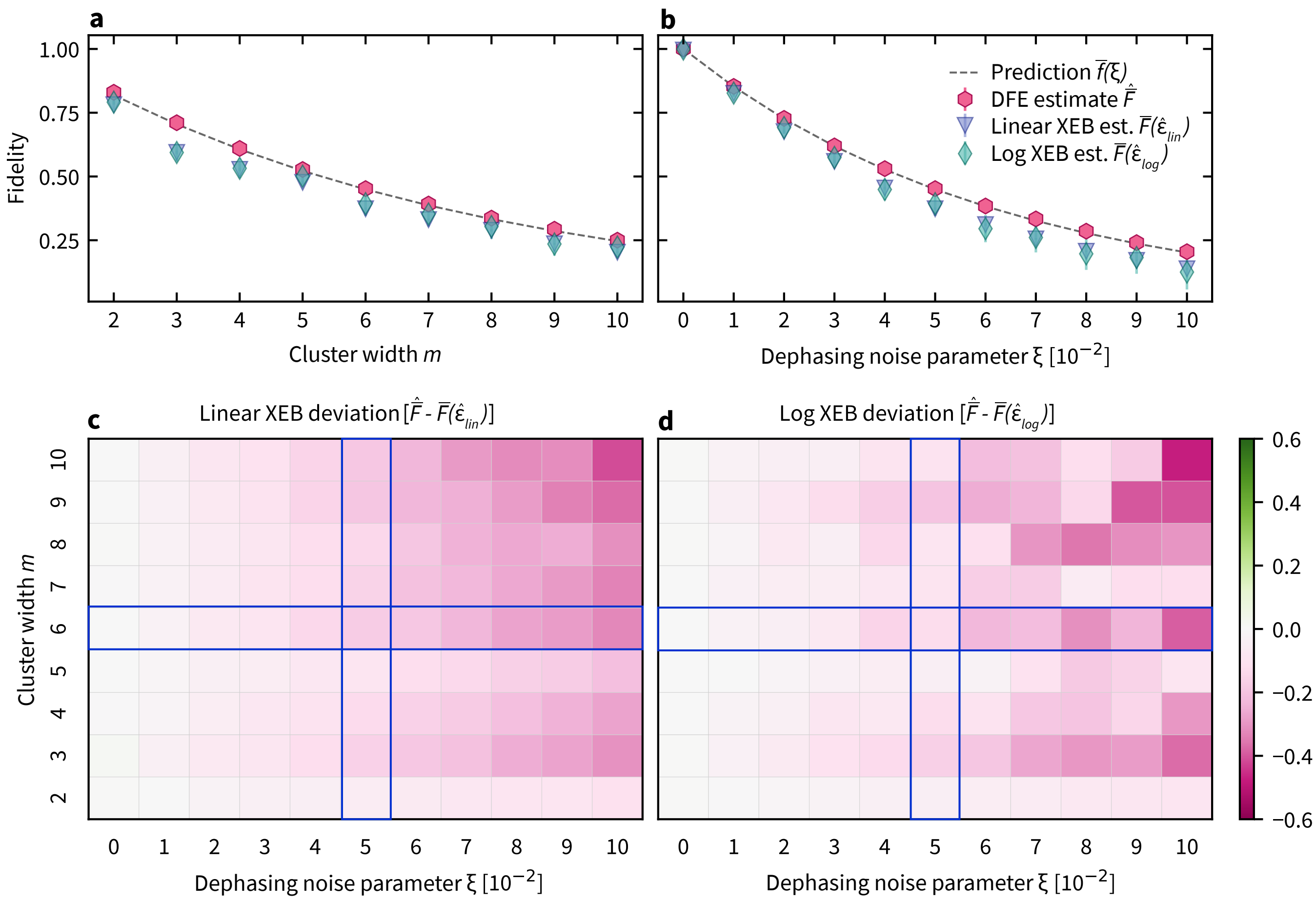

Supplementary Figure 5. **Average fidelity estimation for dephasing noise.** We simulate sampling in the Hadamard basis and DFE for noisy random cluster states of size $2 \times m$ for $m \in \{2, 3, \ldots, 10\}$ with local dephasing noise with parameter ξ after every entangling CZ gate. We sample $K = 10^5$ random circuits and take $M = 50$ samples per circuit. From the classical samples, we compute estimates of the average fidelity $\overline{F}(\hat{\epsilon})$ according to (36) using the linear and logarithmic average XEB fidelity $\overline{f}_{\text{lin}}$ and $\overline{f}_{\text{log}}$ (represented by purple triangles and green diamonds, respectively) and compare them to the DFE estimate $\hat{\overline{F}}$ of the average fidelity (pink hexagons) as well as the prediction (44) by Dalzell *et al.* [20] (dotted line). Error bars are 3σ intervals. (a) Decay of the average fidelity with the width of the cluster state for fixed noise parameter $\xi = 0.05$. (b) Decay of the average fidelity with the noise parameter for a fixed width of the cluster $m = 6$ (blue boxes). (c) Deviation $\overline{F}(\hat{\epsilon}_{\text{lin}}) - \hat{\overline{F}}$ between the fidelity estimate from the linear XEB fidelity and the average fidelity. (d) Deviation $\overline{F}(\hat{\epsilon}_{\text{log}}) - \hat{\overline{F}}$ between the fidelity estimate from the linear XEB fidelity and the average fidelity. .

6. SUPPLEMENTARY NOTE 6 – ERROR ANALYSIS FOR THE MEAN OF MEANS ESTIMATOR

In order to compute the statistical error associated with our estimates of the fidelity and the XEB fidelity, we need to compute the variance of a finite-sample estimator of a random variable A conditioned on a random variable B so that

$$F = \mathbb{E}_{B\sim\mathcal{B}}\left[\mathbb{E}_{A\sim\mathcal{A}}[A|B]\right]. \tag{47}$$

We think of B as the random circuit and A as the samples or random stabilizer values of the random circuit. Concretely, the fidelity estimate, average fidelity estimate (16), and XEB fidelity (18) estimate are obtained as the empirical estimate of the

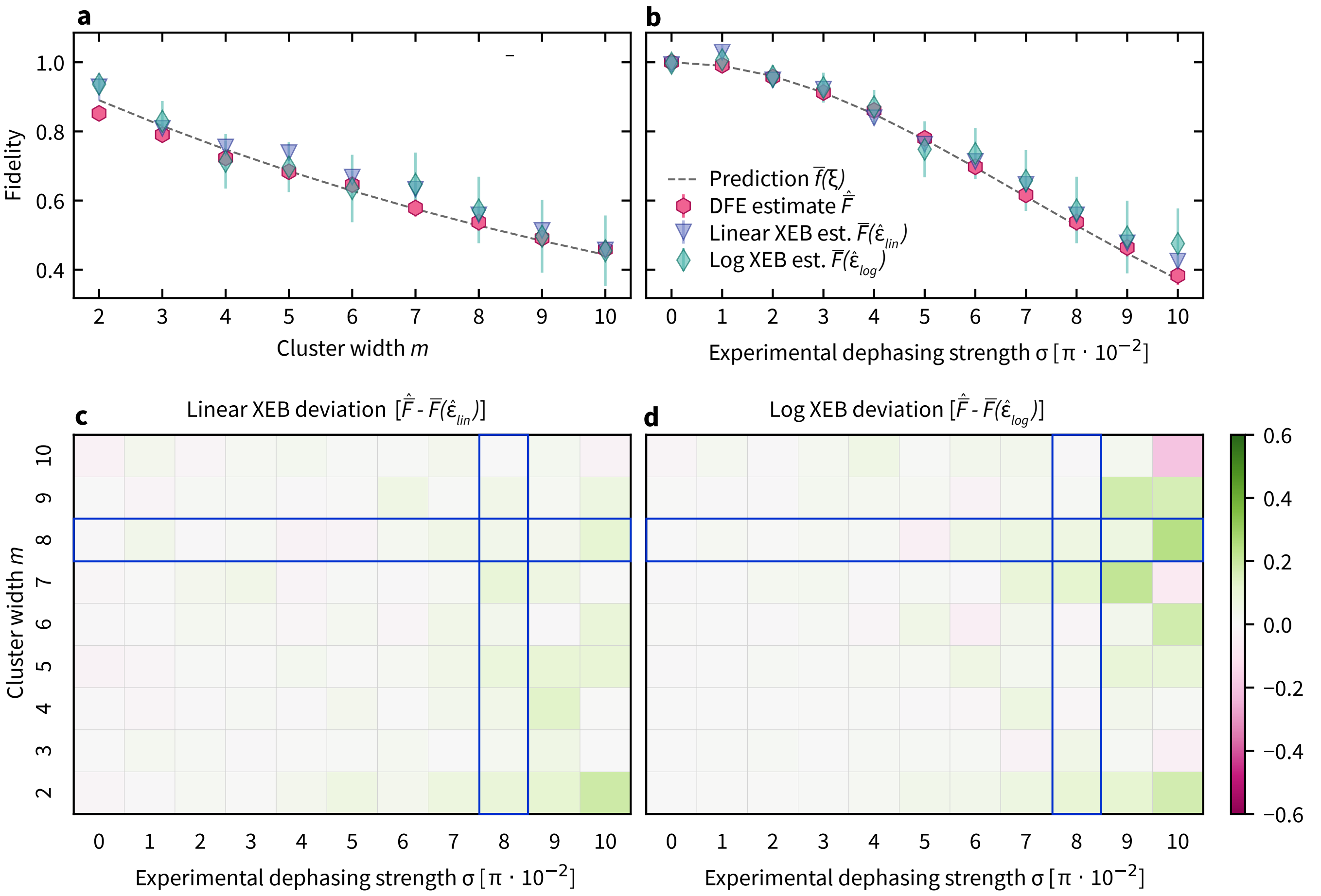

Supplementary Figure 6. **Average fidelity estimation for the noisy experimental circuits.** We simulate sampling in the Hadamard basis and DFE for noisy random cluster states of size $2 \times m$ for $m \in \{2, 3, \dots, 10\}$ with random local Z-rotations with rotation angle drawn from a normal distribution with mean 0 and standard deviation σ inserted in every layer of the experimental circuit; see the Methods section for details. We sample $K = 10^5$ random circuits and take $M = 1$ sample per circuit. From the classical samples, we compute estimates of the average fidelity $\overline{F}(\hat{\epsilon})$ according to (36) using the linear and logarithmic average XEB fidelity $\overline{f}_{\mathsf{lin}}$ and $\overline{f}_{\mathsf{log}}$ (represented by purple triangles and green diamonds, respectively) and compare them to the DFE estimate $\hat{\overline{F}}$ of the average fidelity (pink hexagons) as well as the prediction $\overline{f}(3\gamma/4)$ (44) by Dalzell *et al.* [20] (dotted line). Error bars are 3σ intervals. (a) Decay of the average fidelity with the width of the cluster state for fixed noise strength $\sigma = 0.08\pi$. (b) Decay of the average fidelity with the noise parameter for a fixed width of the cluster $m = 8$ (blue boxes). (c) Deviation $\overline{F}(\hat{\epsilon}_{\mathsf{lin}}) - \hat{\overline{F}}$ between the fidelity estimate from the linear XEB fidelity and the average fidelity. (d) Deviation $\overline{F}(\hat{\epsilon}_{\mathsf{log}}) - \hat{\overline{F}}$ between the fidelity estimate from the linear XEB fidelity and the average fidelity.

expectation values

$$F(\rho, |\psi\rangle\langle\psi|) = \frac{1}{2^N} \sum_{s \in \mathcal{S}} \sum_{\sigma \in \pm 1} \sigma \cdot \mathrm{Tr}[\rho \pi_s^\sigma] \tag{48}$$

$$= \mathbb{E}_{s \sim \mathcal{S}} \left[\mathbb{E}_{\sigma \sim p_{\rho,s}}[\sigma] \right],$$

$$\overline{F} = \frac{1}{2^N} \frac{1}{8^N} \sum_{\beta \in \frac{\pi}{4} \cdot [8]^n} \sum_{s_\beta \in \mathcal{S}_\beta} \sum_{\sigma = \pm 1} \sigma \cdot \langle \pi^\sigma_{s_\beta} \rangle_{\rho_\beta} \tag{49}$$

$$= \mathbb{E}_{\beta, s_\beta \sim \mathcal{S}_\beta} \left[\mathbb{E}_{\sigma \sim p_{\rho_\beta, s_\beta}}[\sigma] \right], \tag{50}$$

$$\overline{f}_{\mathsf{lin}} = \mathbb{E}_{\beta \sim \frac{\pi}{4} \cdot [8]^N} \left[\frac{1}{2^N} \sum_{x \in \{0,1\}^N} Q_\beta(x) \left(2^N P_\beta(x) - 1 \right) \right] \tag{51}$$

$$= \mathbb{E}_{\beta \sim \frac{\pi}{4} \cdot [8]^N} \left[\mathbb{E}_{x \sim Q_\beta} \left[2^N P_\beta(x) - 1 \right] \right],$$

where $p_{\rho,s}(\sigma) = \mathrm{Tr}[\rho\pi_s^\sigma]$ and $s = \pi_s^+ - \pi_s^-$ is a stabilizer of $|\psi\rangle$. We now wish to estimate the variance of a finite-sample estimate of such an expectation value, that is, an estimator

$$\hat{F} = \frac{1}{K}\frac{1}{M}\sum_{i=1}^{K}\sum_{j=1}^{M} A_{i,j}, \tag{52}$$

where K is the number of times the first expectation value is sampled out, and M is the number of times the second expectation value is sampled out, given the result of the first. For instance, to estimate the average bias of a bag of coins with different biases, we draw K coins and flip each drawn coin M times. In this case, $A_{i,j} \in \{0,1\}$ is the outcome of the jth flip of the ith drawn coin.

Very generally, we can compute the variance of such a conditional expectation value using the law of total variance, which states that for two random variables A, B on the same probability space

$$\mathrm{Var}(A) = \mathbb{E}[\mathrm{Var}[A|B]] + \mathrm{Var}[\mathbb{E}[A|B]]. \tag{53}$$

Consider the fidelity estimate. If we let $A_i = \sum_{j=1}^{M} A_{i,j}$ be the random variable, representing the empirical cumulative value of the M measurement outcomes $A_{i,j} \in \{\pm 1\}$ of the stabilizer s_i on the state preparation ρ with associated probability distribution $p_{s_i,\rho}$, then the overall variance is given by $\mathrm{Var}(\hat{F}) = (KM)^{-2}\sum_{i=1}^{K}\mathrm{Var}(A_i)$. To understand the variance $\mathrm{Var}(A_i)$, letting $p_i = p_{s_i,\rho}(+1)$ so that $\mathbb{E}[A_i] = M(2p_i - 1)$, we now invoke the law of total variance, to get

$$\begin{aligned} \mathrm{Var}[A_i] &= \mathbb{E}_{p_i}\left[\mathrm{Var}[A_i|p_i]\right] + \mathrm{Var}_{p_i}\left[\mathbb{E}[A_i|p_i]\right] \\ &= \mathbb{E}[4Mp_i(1-p_i)] + \mathrm{Var}[M(2p_i-1)] \\ &= 4M\mathbb{E}[p_i] - 4M\mathbb{E}[p_i^2] + 4M^2\,\mathrm{Var}[p_i] \\ &= 4M\mathbb{E}[p_i] - 4M(\mathrm{Var}[p_i] + \mathbb{E}[p_i]^2) + 4M^2\,\mathrm{Var}[p_i] \\ &= 4M(\mathbb{E}[p_i](1-\mathbb{E}[p_i])) + 4M(M-1)\,\mathrm{Var}[p_i]. \end{aligned} \tag{54}$$

This yields the overall variance

$$\begin{aligned} \mathrm{Var}[\hat{F}] &= \frac{4}{KM}\left((\mathbb{E}[p_i](1-\mathbb{E}[p_i])) + (M-1)\,\mathrm{Var}[p_i]\right) \\ &= \frac{4}{KM}(\mathbb{E}[p_i](1-\mathbb{E}[p_i])) + \frac{4}{K}\left(1-\frac{1}{M}\right)\mathrm{Var}[p_i]. \end{aligned} \tag{55}$$

Consequently, the variance is asymptotically dominated by the variance over the stabilizers, but in the finite-sample case, there is a trade-off between choosing M and K governed by the specific value of $\mathrm{Var}[p_i]$.

For the case of the (linear and logarithmic) XEB fidelity estimated from K random choices of rotation angles β_i, $i = 1, \ldots, K$ and M samples per β_i, we follow the same reasoning, defining the average estimator $\hat{\bar{f}}_{\mathsf{lin}} = (KM)^{-1}\sum_{i,j=1}^{K,M}(2^N P_{\beta_i}(x_j) - 1)$, and the single-circuit estimator $\hat{f}_{\mathsf{lin}}(Q_\beta, P_\beta) = M^{-1}\sum_{j=1}^{M}(2^N P_{\beta_i}(x_j) - 1)$. We then consider the random variable $A_i = \sum_{j=1}^{M}(2^N P_{\beta_i}(x_j) - 1)$ and $\mathrm{Var}[\hat{\bar{f}}_{\mathsf{lin}}] = (KM)^{-2}\sum_{i=1}^{K}\mathrm{Var}[A_i]$, and find

$$\mathrm{Var}[A_i] = \mathbb{E}[\mathrm{Var}[A_i|\beta_i]] + \mathrm{Var}[\mathbb{E}[A_i|\beta_i]], \tag{56}$$

and estimate the separate terms as

$$\begin{aligned} \mathbb{E}_i[\mathrm{Var}_x[A_i|\beta_i]] &= \mathbb{E}_i[M\,\mathrm{Var}_x[2^N P_{\beta_i}(x) - 1]] \\ &= \mathbb{E}_i[\sum_x Q_{\beta_i}(x)(2^N P_{\beta_i}(x) - 1 - f_{\mathsf{lin}}(Q_{\beta_i}, P_{\beta_i})))^2] \\ &\approx \frac{1}{K}\sum_{i=1}^{K}\sum_{j=1}^{M}(2^N P_{\beta_i}(x_j) - 1 - \hat{f}_{\mathsf{lin}}(Q_{\beta_i}, P_{\beta_i}))^2, \end{aligned} \tag{57}$$

$$\begin{aligned} \mathrm{Var}_i[\mathbb{E}_x[A_i|\beta_i]] &= \mathrm{Var}_i[M f_{\mathsf{lin}}(Q_{\beta_i}, P_{\beta_i})] \\ &\approx M^2\frac{1}{K}\sum_{i=1}^{K}(\hat{f}_{\mathsf{lin}}(Q_{\beta_i}, P_{\beta_i}) - \hat{\bar{f}}_{\mathsf{lin}}(Q_{\beta_i}, P_{\beta_i}))^2. \end{aligned} \tag{58}$$

Overall, we obtain

$$\mathrm{Var}[\hat{\bar{f}}_{\mathsf{lin}}] = \frac{1}{K^2}\frac{1}{M^2}\left(\sum_{i=1}^{K}\sum_{x=1}^{M}(2^N P_{\beta_i}(x) - 1 - f_{\mathsf{lin}}(Q_{\beta_i}, P_{\beta_i}))^2 + M^2\sum_{i=1}^{K}(f_{\mathsf{lin}}(Q_{\beta_i}, P_{\beta_i}) - \bar{f}_{\mathsf{lin}}(Q_{\beta_i}, P_{\beta_i}))^2\right)$$
$$= \frac{1}{KM}\mathbb{E}_\beta[\mathrm{Var}_x[2^N P_\beta(x) - 1]] + \frac{1}{K}\mathrm{Var}_\beta[f_{\mathsf{lin}}(Q_\beta, P_\beta)], \quad (59)$$

which we estimate using the expressions in Eqs. (57) and (58). An analogous expression gives the variance of the logarithmic XEB estimate.

[1] M. Cramer, M. B. Plenio, S. T. Flammia, R. Somma, D. Gross, S. D. Bartlett, O. Landon-Cardinal, D. Poulin, and Y.-K. Liu, *Efficient Quantum State Tomography*, Nat. Commun. **1**, 149 (2010).
[2] D. Hangleiter, M. Kliesch, M. Schwarz, and J. Eisert, *Direct Certification of a Class of Quantum Simulations*, Quantum Sci. Technol. **2**, 015004 (2017).
[3] L. Stockmeyer, The Complexity of Approximate Counting, in *Proceedings of the Fifteenth Annual ACM Symposium on Theory of Computing* (ACM, 1983) pp. 118–126.
[4] D. Hangleiter, *Sampling and the Complexity of Nature*, Ph.D. thesis, Freie Universität, Berlin (2021), arxiv:2012.07905.
[5] D. Hangleiter and J. Eisert, *Computational Advantage of Quantum Random Sampling*, Rev. Mod. Phys. **95**, 035001 (2023).
[6] J. Bermejo-Vega, D. Hangleiter, M. Schwarz, R. Raussendorf, and J. Eisert, *Architectures for Quantum Simulation Showing a Quantum Speedup*, Phys. Rev. X **8**, 021010 (2018).
[7] K. Fujii and T. Morimae, *Commuting Quantum Circuits and Complexity of Ising Partition Functions*, New J. Phys. **19**, 033003 (2017).
[8] S. Aaronson and L. Chen, Complexity-Theoretic Foundations of Quantum Supremacy Experiments, in *32nd Computational Complexity Conference (CCC 2017)*, Leibniz International Proceedings in Informatics (LIPIcs), Vol. 79, edited by R. O'Donnell (Schloss Dagstuhl–Leibniz-Zentrum fuer Informatik, Dagstuhl, Germany, 2017) pp. 22:1–22:67.
[9] B. Barak, C.-N. Chou, and X. Gao, Spoofing Linear Cross-Entropy Benchmarking in Shallow Quantum Circuits, in *12th Innovations in Theoretical Computer Science Conference (ITCS 2021)*, Leibniz International Proceedings in Informatics (LIPIcs), Vol. 185, edited by J. R. Lee (Schloss Dagstuhl–Leibniz-Zentrum für Informatik, Dagstuhl, Germany, 2021) pp. 30:1–30:20, arxiv:2005.02421.
[10] S. T. Flammia and Y.-K. Liu, *Direct Fidelity Estimation from Few Pauli Measurements*, Phys. Rev. Lett. **106**, 230501 (2011).
[11] F. Arute *et al.*, *Quantum Supremacy Using a Programmable Superconducting Processor*, Nature **574**, 505 (2019).
[12] Q. Zhu *et al.*, *Quantum Computational Advantage via 60-Qubit 24-Cycle Random Circuit Sampling*, Science Bulletin **67**, 240 (2022).
[13] J. Choi, A. L. Shaw, I. S. Madjarov, X. Xie, R. Finkelstein, J. P. Covey, J. S. Cotler, D. K. Mark, H.-Y. Huang, A. Kale, H. Pichler, F. G. S. L. Brandão, S. Choi, and M. Endres, *Preparing Random States and Benchmarking with Many-Body Quantum Chaos*, Nature **613**, 468 (2023).
[14] S. Boixo, S. V. Isakov, V. N. Smelyanskiy, R. Babbush, N. Ding, Z. Jiang, M. J. Bremner, J. M. Martinis, and H. Neven, *Characterizing Quantum Supremacy in Near-Term Devices*, Nat. Phys **14**, 595 (2018).
[15] S. Aaronson and S. Gunn, *On the Classical Hardness of Spoofing Linear Cross-Entropy Benchmarking*, (2020), arxiv:1910.12085.
[16] A. Bouland, B. Fefferman, C. Nirkhe, and U. Vazirani, *On the Complexity and Verification of Quantum Random Circuit Sampling*, Nat. Phys **15**, 159 (2019).
[17] J. Haferkamp, D. Hangleiter, A. Bouland, B. Fefferman, J. Eisert, and J. Bermejo-Vega, *Closing Gaps of a Quantum Advantage with Short-Time Hamiltonian Dynamics*, Phys. Rev. Lett. **125**, 250501 (2020).
[18] A. M. Childs, D. W. Leung, and M. A. Nielsen, *Unified Derivations of Measurement-Based Schemes for Quantum Computation*, Phys. Rev. A **71**, 032318 (2005), arxiv:quant-ph/0404132.
[19] D. Hangleiter, J. Bermejo-Vega, M. Schwarz, and J. Eisert, *Anticoncentration Theorems for Schemes Showing a Quantum Speedup*, Quantum **2**, 65 (2018).
[20] A. M. Dalzell, N. Hunter-Jones, and F. G. S. L. Brandão, *Random Quantum Circuits Transform Local Noise into Global White Noise*, Commun. Math. Phys. **405**, 78 (2024).
[21] N. Usher and D. E. Browne, *Noise in One-Dimensional Measurement-Based Quantum Computing*, (2017), arxiv:1704.07298.
